# Microstereolithography-Based Fabrication of Anatomically Shaped Beta-Tricalcium Phosphate Scaffolds for Bone Tissue Engineering

**DOI:** 10.1155/2015/859456

**Published:** 2015-10-04

**Authors:** Dajiang Du, Teruo Asaoka, Makoto Shinohara, Tomonori Kageyama, Takashi Ushida, Katsuko Sakai Furukawa

**Affiliations:** ^1^Department of Orthopaedic Surgery, Sino-Russian Institute of Hard Tissue Development and Regeneration, Harbin Medical University, Nangang, Harbin 150086, China; ^2^Department of Bioengineering, University of Tokyo, Bunkyō, Tokyo 113-8656, Japan; ^3^Department of Mechanical Engineering, Tokyo Denki University, Adachi, Tokyo 101-8457, Japan; ^4^Center for Disease Biology and Integrative Medicine, University of Tokyo, Bunkyō, Tokyo 113-8656, Japan

## Abstract

Porous ceramic scaffolds with shapes matching the bone defects may result in more efficient grafting and healing than the ones with simple geometries. Using computer-assisted microstereolithography (MSTL), we have developed a novel gelcasting indirect MSTL technology and successfully fabricated two scaffolds according to CT images of rabbit femur. Negative resin molds with outer 3D dimensions conforming to the femur and an internal structure consisting of stacked meshes with uniform interconnecting struts, 0.5 mm in diameter, were fabricated by MSTL. The second mold type was designed for cortical bone formation. A ceramic slurry of beta-tricalcium phosphate (*β*-TCP) with room temperature vulcanization (RTV) silicone as binder was cast into the molds. After the RTV silicone was completely cured, the composite was sintered at 1500°C for 5 h. Both gross anatomical shape and the interpenetrating internal network were preserved after sintering. Even cortical structure could be introduced into the customized scaffolds, which resulted in enhanced strength. Biocompatibility was confirmed by vital staining of rabbit bone marrow mesenchymal stromal cells cultured on the customized scaffolds for 5 days. This fabrication method could be useful for constructing bone substitutes specifically designed according to local anatomical defects.

## 1. Introduction

The repair of bone defects by tissue grafting is a major clinical application of cell-based therapies in modern orthopedic and maxillofacial surgery. However, conventional harvesting of bone for autografts is invasive and can damage the donor-site, while allografts present obvious risks of rejection and viral transmission [1]. A superior option is to seed smaller bone fragments or cultured stem cells onto scaffolds with osteoconductive or osteoinductive properties [2]. Calcium phosphate grafts have been applied for bone reconstructive surgery [3]. A close fit between the defect site and the artificial bone substrate is important for improving bone healing and stress transduction [4], but it has proven difficult to tailor ceramic scaffolds to fit specific defects. For this reason, ceramics are generally used as granules or beads instead of single structures [5], which makes the material difficult to use when the anatomic geometry of the repair site is of major concern for the reconstructive outcome, such as for maxillofacial surgery. Therefore, ceramic scaffolds customized according to the external anatomic contours of the defect site could improve clinical outcome.

Computer-aided techniques play an increasingly important role in modeling, designing, and manufacturing, including the production of biological tissue substitutes. Microstereolithography (MSTL) is a micromanufacturing process used to fabricate three-dimensional (3D) structures conforming to predetermined specifications. The process achieves superior replication of design specifications compared to all other 3D printing technologies and so is a promising methodology for producing scaffolds with complex 3D microstructures for tissue engineering. However, direct MSTL-based scaffold fabrication requires biodegradable photocurable biomaterials, which is currently very limited [6–11]. In contrast, MSTL-based indirect SFF technology can be employed to produce 3D biocompatible scaffolds by a lost-mold shape forming process using MSTL to obtain a negative mold, filled with castables, and subsequently remove the mold to get a 3D scaffold [12]. By using MSTL technology to fabricate a resin scaffold mold, the scaffolds can be formed subsequently using a variety of biomaterials with known internal microstructure, durability, and biocompatibility, such as hydroxyapatite [12], calcium phosphate [13], and chitosan/gelatin [14].

Both chemical dissolution [15] and thermal decomposition have been used for the removal of the mold while keeping scaffold shape and biomaterial properties. However, chemical dissolution requires large concentrations of chemically resistant binders that may weaken the sintered body [16, 17]. During the thermal decomposition, the thermal contraction mismatch between the scaffold and mold can distort or fracture the scaffold [18]. Although wax is conventionally used for lost-mold processing and results in little stress on the scaffold during removal, wax was currently only used for the fabrication of bioscaffolds with simple geometries and its resolution was limited [19–21]. In contrast, gelcasting is a molding technique using a concentrated ceramic or metallic powder slurry containing monomers that polymerize to form a sturdy and durable yet precisely shaped cross-linked gel scaffold after casting [21–23]. Room temperature vulcanization (RTV) silicone rubber has low viscosity, good flowability, low shrinkage, and high temperature resistance. We predicted that the slurry produced by mixing a ceramic powder into the RTV silicone will both conform to the resin mold and, once set by polymerization, show little shrinkage or damage during the lost-mold process, thereby maintaining the as-molded geometry with high resolution. Thus, this gelcasting indirect MSTL-based technique could yield bone scaffolds with complex anatomical shapes and highly defined internal structures to match bone defects for hard tissue reconstruction.

In this study, a segment of rabbit femur was used as a bone-defect model for reconstruction and *β*-tricalcium phosphate (*β*-TCP) was chosen as ceramic material for scaffolds. We established a gelcasting indirect MSTL fabrication process and fabricated *β*-TCP scaffolds shaped according to CT measurements of the bone and with a well-designed internal lattice network for stem cell growth. The scaffolds were fabricated either with or without a cortical bone structure to investigate if such a biomimetic structure could enhance mechanical strength and reflect fine surface details. Assessment of morphology and mechanical properties of both scaffold types revealed accurate shape matching between mold and scaffold, as designed, and scaffold strength with the range of cancellous bone. Rabbit's bone marrow mesenchymal stromal cells (BM-MSCs) were viable after 5 days in vitro in these scaffolds, confirming biocompatibility.

## 2. Materials and Methods

### 2.1. Customized Scaffold Design according to CT Data

**Figure 1:**
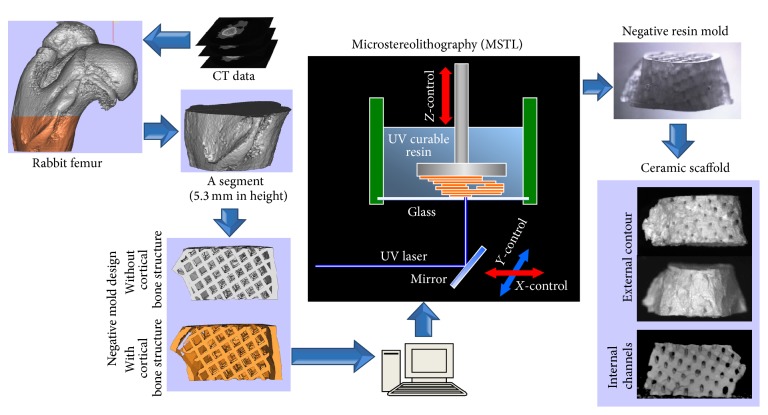
The schematic illustration for the main steps in design and fabrication of anatomically shaped ceramic scaffolds with or without a cortical bone structure by microstereolithography (MSTL).

The main steps in scaffold design and fabrication are shown schematically in Figure 1. As the target template (i.e., a bone-defect model), an anatomical 3D surface model was reconstructed according to the CT data scanned from the rabbit proximal femur and converted into stereolithography (STL) file format using a commercially available computer software program (Mimics 10.0; Materialise Co. Ltd., Leuven, Belgium).

A 3D orthogonal network of interpenetrating, round pores (diameter = 500 *μ*m) was designed for the internal structure. Then, a 3D STL model of the mold with inverse morphology of the target ceramic scaffold was obtained. In order to mimic the nature bone and improve the mechanical properties of the scaffold, a second near-identical mold was also designed allowing for cortical bone structure (0.5 mm in thickness). The top and bottom of this mold were designed as open faces together with sealed sides to facilitate filling of the viscous ceramic slurry and to allow gas to escape from the mold. Magic's 10 (Materialise Co. Ltd., Leuven, Belgium) was used in this process.

### 2.2. Microstereolithography-Based Indirect Fabrication Scaffold

A microstereolithography system equipped with a helium-cadmium ultraviolet laser (LC-500, Melles Griot, Carlsbad, CA) was used to produce the resin models. The laser power was adjusted to 25 *μ*W using neutral density filters. “Slice” contour data were generated from the 3D STL data to guide the laser beam in the *X*-*Y* plain for hardening of the UV-curable resin. The UV laser path (V*x*-*y* = 3 mm/s) was controlled by a computer-connected reflection mirror. Intricate 3D 33 structures were fabricated by stacking up cross-sectional resin slices (slice thickness ~80 *μ*m, V*z* = 1 mm/s) according to the predesigned STL data. The negative UV-cured resin molds fabricated by microstereolithography were sonicated in 80% Ethanol for 30 min to remove the unsolidified resin.

**Figure 2:**
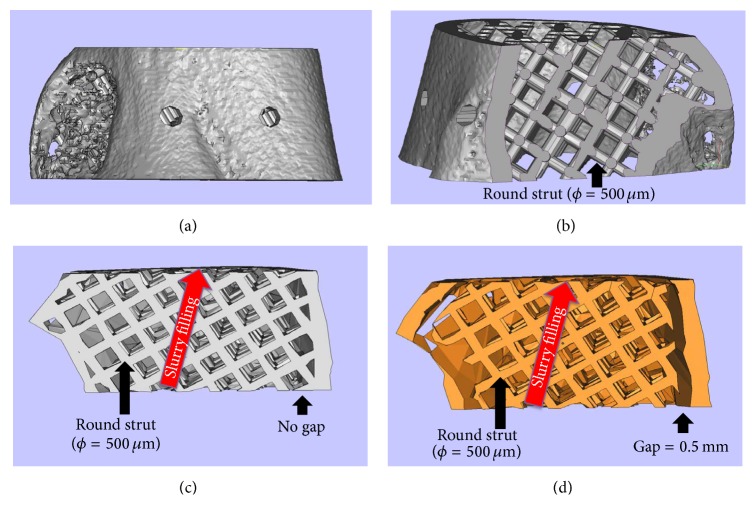
The design of negative mold for the scaffolds without and with cortical bone structure. (a) The external shape according to the anatomical contour of the femoral segment and (b) the design of the internal strut networks, which have the inverse morphology of the channels in the scaffolds; (c) and (d) show the section view of the designed negative mold without and with cortical structure design, respectively. A gap of 0.5 mm between outer shell and lattice is set to introduce a cortical bone-like structure.

Room temperature vulcanization silicone was used to make the *β*-TCP slurry (70 wt% solids). After removal of unsolidified resin by washing, *β*-TCP slurry was filled completely into the fabricated resin mold in the direction as shown in Figure 2. The scaffold samples were allowed to set at room temperature overnight for the silicone to cross-link completely. Subsequently, the samples were sintered at 1,500°C. A dwell time was added at 600°C for the removal of the mold resin.

### 2.3. Morphological Study

Stereomicroscopy was employed to assess outer shape and pore microstructure of the molds and scaffolds. CT examinations were performed using a microfocus X-ray CT system (100 *μ*A, 90 kV). The heights of the molds and the sintered scaffolds were measured with digital calipers. The mean cross-sectional pore diameter (± standard deviation) and the porosity were calculated by measuring 30 pores/sample from reconstructed micro-CT scans.

### 2.4. Mechanical Study of the Ceramic Scaffold by MSTL

The mechanical testing was performed using a universal testing machine. All samples were compressed at 1 mm/min. After the ultimate compressive force was measured, the ultimate compressive strength was calculated. Young's modulus was then derived from the stress-strain curve.

### 2.5. Cell Viability Analysis

Rabbit's BM-MSCs were isolated and expended as previously described [24]. The BM-MSCs were seeded into each scaffold at 3.0 × 10^6^ in 100 *μ*L in low-glucose Dulbecco's-modified Eagle's medium (DMEM; GIBCO, Grand Island, NY) containing 10% fetal bovine serum (GIBCO) and 1% penicillin-streptomycin. Culture media were exchanged every 2 days. After 5 days of culture, samples were harvested and stained using a live/dead cell double staining kit (DOJINDO, Kumamoto, Japan). After washing by phosphate-buffered saline (PBS), each sample was immersed in calcein-AM/propidium iodide (PI) dual staining solution and incubated for 15 min at 37°C. The samples were examined using a fluorescence stereomicroscope as previously described [25].

### 2.6. Statistics

All values are presented as mean ± standard deviation. Differences among experimental groups were assessed by two-tailed Student's *t*-test. A *p* < 0.05 was considered statically significant.

## 3. Results

3D STL data derived from the CT images were used for computer-assisted microstereolithography (3D printing) of a resin mold with an internal lattice structure (Figure 1). In turn, the resin mold was used for gelcasting of a ceramic scaffold. The outer shape of the fabricated scaffold was identical to the anatomical structure of the scanned femur, and an interconnected channel network with round channels (diameter = 500 *μ*m) at 45° to the ceramic slurry filling direction was retained after mold burnout. The STL data for mold fabrication were derived as shown in Figure 2. To introduce a cortical bone-like structure, modified molds were also constructed with a gap of 0.5 mm between outer shell and lattice (Figure 2(d)).

**Figure 3:**
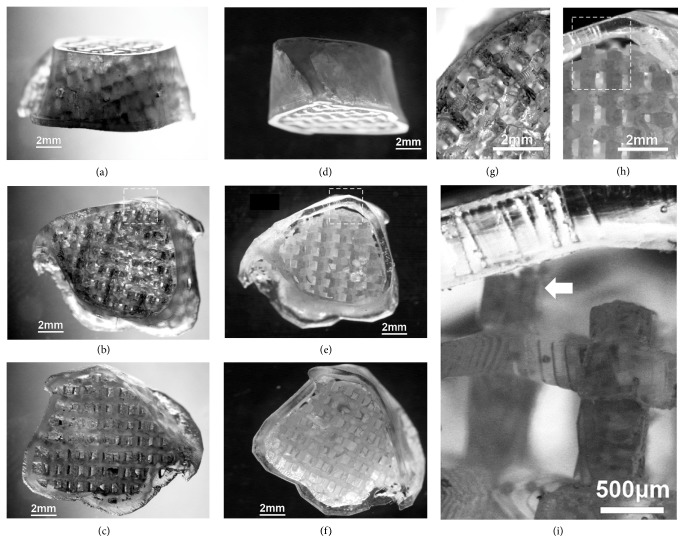
The negative UV-cured resin mold fabricated by microstereolithography. (a)–(c): the mold for scaffolds without cortical design; (d)–(f): the mold for scaffolds with cortical design. (a) and (d) are the side views; (b) and (e) are the top views; (c) and (f) are the bottom views. (g) and (h) showed the location of boxes in (b) and (e) by larger magnification. (i) showed the edge design for producing the cortical bone-like structure, where the box area in (h) was amplified. The white arrow indicates one of the lattice struts connecting between the internal lattice network and the outer shell.

Figure 3 shows the detailed microscopy structures of sample UV-cured resin molds produced by MSTL. The lattice network with inverse morphology to the internal channels in the scaffold exhibited consistent geometry (Figures 3(b) and 3(c)). A gap between the outer shell and internal lattice network was incorporated in the design to allow for cortical bone formation (Figures 3(e) and 3(h)). A few lattice struts were designed to connect the internal and outer parts in the cortical design mold (Figure 3(i)). The surface of the lattice struts in the mold was highly textured due to the thin-layer MSTL process (Figure 3(i)). Thus, these molds share many of the morphological characteristics of previous molds produced by rapid prototyping [26, 27].

**Figure 4:**
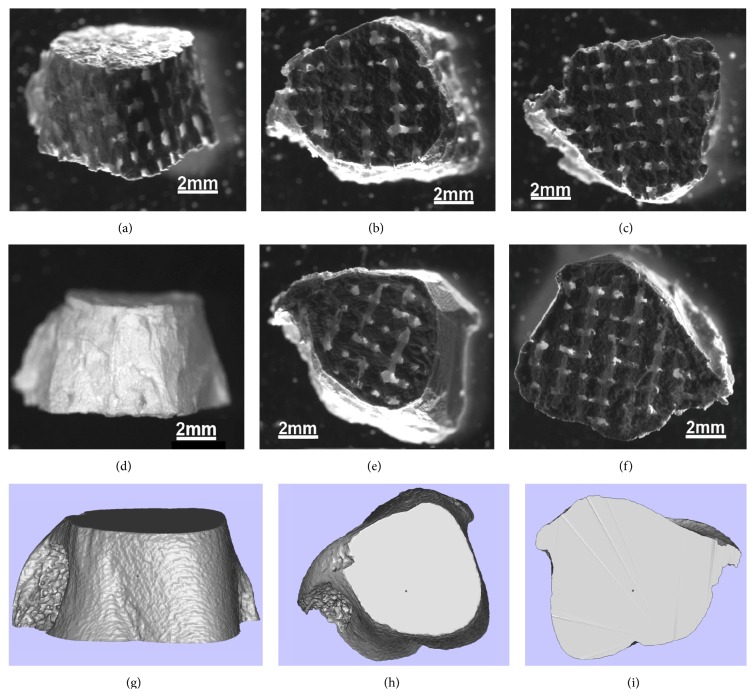
The anatomically shaped ceramic scaffolds fabricated by indirect rapid prototyping technique. (a)–(c) are the scaffolds without cortical design; (d)–(f) are the scaffolds with cortical design; (g)–(i) are the STL data of the template bone. (a), (d), and (g) are the side view; (b), (e), and (h) are the top view; (c), (f), and (i) are the bottom view.

After filling the mold with ceramic slurry, sintering, and mold burnout, *β*-TCP scaffolds both with and without cortical structure were produced. The sintered scaffolds retained the external bone contour specified by STL data (Figure 4). A bone cortex-like structure was produced by filling the gap incorporated in the modified “cortex design” molds (Figures 4(d)–4(f)), and these modified scaffolds exhibited more anatomical details than the noncortical design scaffolds.

**Figure 5:**
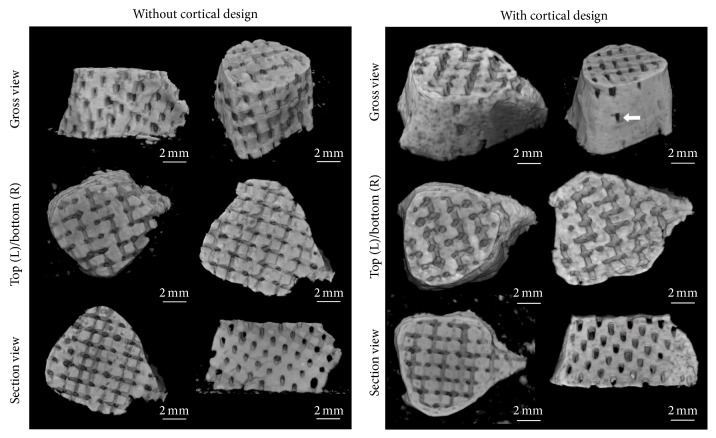
The reconstruction images from micro-CT scanning of the sintered scaffolds. The white arrow indicates nutrient vessel-like structures resulting from the removal of the struts connecting the internal lattice and outer shell.

A micro-CT study of the sintered scaffolds confirmed reproduction of the external shape of the template bone (Figure 5). In addition to preserving bone surface morphology, the surfaces of the cortex design scaffolds exhibited nutrient vessel-like structures (white arrow, Figure 5), resulting from the removal of the out resin lattice struts in the cortex design resin mold. Reconstructed images showed that both the cortex design and noncortex scaffolds had a consistent and continuous internal porous network without occlusions.

Morphometric measurements (Table 1) showed that the resin molds (both types) were produced as designed, with only a 0.04 mm difference in height (5.31 mm as designed versus 5.33 or 5.35 mm). However, the ceramic scaffolds shrank by about 9% on average during sintering. The cross-sectional diameter of the pores as measured by micro-CT was 0.45 ± 0.03 mm for the scaffolds without cortical design and 0.44 ± 0.02 mm for those with cortical design, only ~10% smaller than the design diameter (0.50 mm) and well within the range required for bone tissue engineering applications [28]. As the cortical structure occupied some of the lattice space (Figure 2(d)), the porosity of scaffolds with cortical structure was ~31% lower than that of scaffolds without cortical structure.

**Figure 6:**
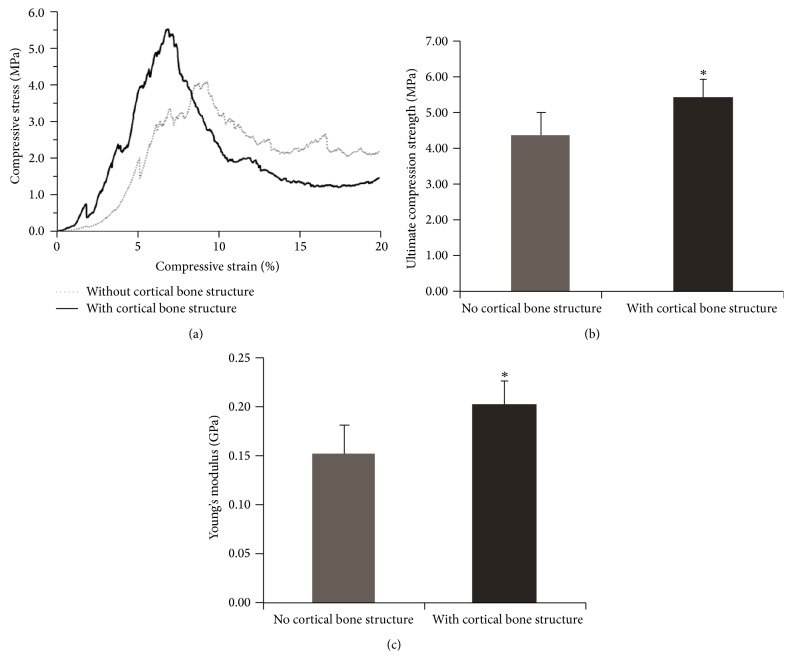
The mechanical properties of the sintered ceramic scaffolds. (a) The stress-strain curve; (b) ultimate compression strength; (c) Young's modulus. Error bars represent standard deviation (SD), *n* = 7. The asterisk (*∗*) indicates a statistically significant difference between the static group and the perfusion group (*p* < 0.05).

The stress-strain curve showed that the compressive stress on sintered scaffolds gradually increased with compress strain until load drop indicative of ultimate compression strength (Ult. Comp. strength) (Figure 6(a)). Both Ult. Comp. strength and Young's modulus were higher in the scaffolds with cortical structure (*n* = 7, *p* < 0.05) (Figures 6(b) and 6(c)), suggesting that the thicker cortex-like structure enhanced scaffold strength and prevented damage to the porous internal structure. The Ult. Comp. strength of both scaffold types was comparable to trabecular bone (0.6−15 MPa [29]; thus well suited for bone tissue engineering applications [11].

**Figure 7:**
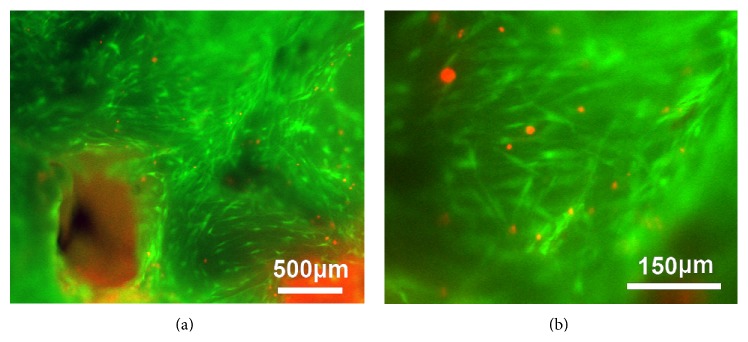
Fluorescence microscopy images of the rabbit BMSCs cultured on the ceramic scaffolds for 5 days. Calcein-AM/PI double staining was performed to study the cell viability. (a) was observed by 4x objective lens and (b) was observed by 10x objective lens (green, living cell; red, apoptotic cell).

By Calcein-AM/PI staining, we tested scaffold biocompatibility by evaluating the viability of rabbit BM-MSCs after culturing for 5 days (Figure 7). Many viable (calcein-stained) rabbit's BM-MSCs were attached on the porous surface of the customized scaffolds with few (PI-stained) apoptotic cells scattering among them. Further observation with higher magnification fluorescence microscopy revealed that the cells on the pore surface took on the stretched or spindle-like shape typical of cultured BM-MSCs. Therefore, biocompatibility criteria were satisfied.

## 4. Discussion

We fabricated ceramic scaffolds with the external shape and internal porous structure specified by a resin mold designed based on bone CT imaging and constructed using microstereolithography. Moreover, these scaffolds demonstrated good biocompatibility for growth of bone marrow mesenchymal stromal cells. This two-step (indirect) MSTL-based method allowed for the construction of anatomically complex scaffolds using ceramic material (beta-tricalcium phosphate) of known malleability and biocompatibility and so may facilitate the rapid production of scaffolds that conform to specific bone defects for optimal surgical repair.

MSTL creates complex 3D structures by curing resin using UV lasers, so direct fabrication of scaffolds would require UV-curable biomaterials rather than biomaterials with known biocompatibility and osteoinductive capacity [4]. To overcome this limitation, we used MSLT to design and create resin molds for beta-tricalcium phosphate scaffolds. However, differences in thermal response between the resin and scaffold material can create cracks in the scaffold during sintering [30]. Indeed, we attained only small ceramic particles (rather than complete scaffolds) in preliminary experiments using traditional water-based formulations such as polyvinyl alcohol as the slurry binder (data not shown), likely, because the ceramic scaffold shrank during sintering and was therefore broken up by the resin lattice struts. We tested RTV silicone rubber as a binder owing to its low viscosity and good flowability, which would facilitate complete filling of the mold. In addition, we also speculated that the low shrinkage and high temperature resistance of RTV would help overcome the thermal mismatch between the resin mold and the raw ceramic material (*β*-TCP). After sintering, the customized ceramic scaffolds exhibited the anatomical details incorporated into the mold design and the specified internal lattice structure without obvious cracking as revealed by microscopic and micro-CT examination (Figures 4 and 5). Preservation of mold design structure may have been aided by inclusion of a dwell time at 600°C during heating, thereby minimizing shrinkage and damage of the ceramic before the resin mold became soft. Although the mechanical strength of these scaffolds was still within the range of trabecular bone and thus insufficient for use in load-bearing regions (Figure 6), this two-step process may be amenable to the use of stronger binder materials for gelcasting. Moreover, strength was augmented by a modified mold including an external gap for formation of cortex-like bone.

A higher solid loading of ceramic powder (%wt) in the slurry will enhance the mechanical strength of the sintered ceramic scaffold, but the viscosity would also increase dramatically, impeding complete mold filling and possibly damaging the delicate resin strut network. To avoid poor mold filling and strut damage, the internal porous structure was designed at 45° (Figure 2) rather than the traditional 90° to the expected slurry filling direction (top to bottom). This choice was based on our preliminary flow simulation experiments, which demonstrated that this design will distribute the fluid more homogenously in the network under a given inlet pressure (data not shown). Therefore, this strut network design may have also facilitated even dispersion of the ceramic slurry into the molds without significant mold damage.

The scaffolds with cortical design better reflected the anatomical details of the template bone compared to scaffolds without cortical design (Figure 4) and even exhibited nutrient vessel-like structures on the surface (Figure 5). In addition, this cortical design also enhanced scaffold mechanical strength (Figure 6). Therefore, the mechanical properties of artificial bone grafts can be modulated by geometric design elements like cortical bone-like structure. However, incorporation of cortex-like bone would also restrict blood vessel invasion from host tissue and reduce space for cell growth. Therefore, it may be useful to combine the cortical and noncortical design within a single scaffold or use multiple fitted scaffolds with and without cortical design for certain graft applications.

Dual calcein/PI staining showed that rabbit BM-MSCs could be cultured successfully on the customized scaffolds (Figure 7). The cells showed typical BM-MSC morphology, indicating that these scaffolds had no notable adverse effects on cell growth or phenotype in this short-term in vitro study. As cells may undergo apoptosis in the internal scaffold after prolonged static culture, in our future work, we will employ hydrodynamic culture system [25, 31] to evaluate the biocompatibility for a prolonged culture period. It is also necessary to evaluate the customized scaffolds using a large animal model with a normal immune system to confirm their regenerative performance in vivo.

## 5. Conclusions

Anatomically shaped *β*-TCP ceramic scaffolds were fabricated successfully by a novel gelcasting indirect MSTL approach. Both the external anatomic shape of the template bone and the designed internal network were preserved after mold burnout. Cortical structure could be introduced by modifying the mold and this strengthened the scaffold. In addition, these *β*-TCP scaffolds exhibited good biocompatibility. Therefore, it may be possible to treat bone defects with geometrically tailored bone substitutes.

## Figures and Tables

**Table 1:** The height, pore diameters (cross section), and the porosity of ceramic samples.

Scaffold types	The height of the customized TCP scaffolds				Pore diameters (mm)	Porosity (vol%)
	Designed (mm)	Resin mold (mm)	Sintered (mm)	Shrinkage (%)		
Without cortical bone (*n* = 7)	5.31	5.33 ± 0.09	4.90 ± 0.17	8.02 ± 3.48	0.45 ± 0.03	26.57 ± 1.05
With cortical bone (*n* = 8)	5.31	5.35 ± 0.05	4.86 ± 0.24	9.11 ± 4.56	0.44 ± 0.02	18.25 ± 1.69
